# Wind Turbine Blade Material Behavior in Abrasive Wear Conditions

**DOI:** 10.3390/polym16243483

**Published:** 2024-12-13

**Authors:** Cristian Muntenita, Larisa Titire, Mariana Chivu, Geanina Podaru, Romeo Marin

**Affiliations:** 1 Faculty of Engineering, “Dunărea de Jos” University, 800008 Galati, Romania; cristian.muntenita@ugal.ro (C.M.); geanina.podaru@ugal.ro (G.P.); romeo.marin@ugal.ro (R.M.); 2 Faculty of Entrepreneurship, Engineering and Business Management, National University of Science and Technology Politehnica, 060042 Bucharest, Romania; mariana.chivu0608@upb.ro

**Keywords:** wind turbine blade, polymeric composite material, abrasive wear

## Abstract

The wind turbine blades are exposed, during functioning, to the abrasive wear generated by the impact with air-borne sand particles. In this work, samples of a commercial wind turbine blade, made of a multi-layered composite material, are subjected to abrasive wear tests, using an air streamed wearing particles test rig. Following the analysis of the tests’ results was found that the only protection against failure of the blade by abrasive damage is the surface layer. After its’ penetration, the layers below are quickly destroyed, leading to the blade destruction. The investigation of the main abrasive wear influencing factors—particles’ speed and acting time, showed that the particles’ speed is the most important. To prove that an artificial neural network-based model was used. Also, a method for improvement of the blade resistance to abrasive wear is proposed, consisting of applying on the blade’s surface of a polymeric foil. This offers supplementary protection of the surface layer, delaying its degradation. The tests performed on the protected samples prove the validity of the proposed method. Overall, the work showed the weakness of the blades’ resistance in case of working in abrasive wear conditions and identified an improving method.

## 1. Introduction

One of the solutions to reduce greenhouse gas emissions, the main contributors to climate change in recent years, is the drastic decrease in fossil fuel consumption. This reduction, in the context of the exponential increase in energy demand worldwide, becomes possible through the identification and exploitation of renewable energy sources, capable of ensuring the consumption needs under sustainability conditions. The European Parliament issued a series of directives, which regulate the obtaining of energy from renewable sources, starting in 2001—Directive 2001/77/EC, regarding the obtaining of the electrical energy [1] continuing with Directive 2003/30/EC, regarding the use of the bio-fuels [2] and Directive 2009/28/EC [3], establishing national objectives until 2020. In 2021, the European Green Deal (Delivering the European Green Deal) [4] is established, which aims to bring Climate neutral Europe by 2050.

In order to meet the imposed requirements, it is necessary to expand the use of renewable energies. Among these, wind energy takes an important place. This energy is generated by air currents that circulate between different geographical areas and its exploitation is carried out with the help of the wind turbines, placed both in onshore and offshore areas. Taking into account that the most exposed component of a wind turbine is the propeller, in order to obtain high efficiency, this one must comply with high criteria, regarding both mechanical resistance and also, high resistance to environmental factors’ action. The solution used on a large scale for these requirements is the use of composite materials. As Mishnaevsky et al. [5] shown, the matrix used for these composites consists of thermoset polymer materials, and more than 80% of current materials use epoxy resin matrices. The main qualities are the possibility of processing at ambient temperature and low viscosity. Glass fibers are some of the main reinforcements used in epoxy resin composites for wind blades. Following Thomas et al. [6], the strength of the composite is determined both by the strength of the fibers and by their volume percentage in the final material.

Taking into account the length of the wind turbine blades and the operating speed, the tangential speed can reach high values, Zhang et al. [7], exceeding 150 m/s (540 km/h). Under these conditions, a series of mechanical phenomena occur that can lead to significant degradation of the blades. Thus, in addition to affecting the aerodynamic performance, due to the shape of the contact surfaces with the air currents, the mechanical resistance of the blade is also affected.

One of the most aggressive environmental factors is abrasive wear, due to the contact of the blade with high-speed particles like sand, dust, etc. This type of degradation can be avoided by adding hard materials, in the form of powders, to the blade composite, as Mathavan et al. shown in [8], or by covering the blade with wear-resistant protective layers, following the recommendation of Mishnaevsky et al. [9]. Wear also occurs in the case of blade contact with raindrops, mainly due to the blade’s high speed, generating cracks and detachments of the material, as Slot et al. specified in [10]. The simple development of new composite materials is not enough if it is not doubled by experimental studies, the results of which allow the optimization of these materials, taking into account the demands they are subjected to during operation. As a consequence, the blade composite material must be designed based on accurate tests, in order to improve abrasive wear resistance. There are specific testing methodologies used for blade resistance of blade materials. Sorensen et al. present in [11] detailed procedures for structural testing, meanwhile Gee et al. [12] specify the main aspects of abrasive wear testing.

Several scholars investigated the wind blades’ materials. Dathu et al. [13] and Patnaik [14], looked to find the wear evolution, in several abrasive working conditions, for different composite materials recipes. Their goal is to propose new materials and test these comparing them with regular ones in order to prove higher resistance properties. There is a gap in this area, the researchers disregard how industrial wind blades, already being in work, behave when abrasive wear is present. However, there is a lack of information on how the composite layers react to the wear evolution.

The present work aims to identify the occurrence and evolution of degradation due to erosion that occurs if the industrial wind blades’ materials come in contact with abrasive particles, disregarding structural damage caused by mechanical fatigue, by the exceed of the permissible mechanical stresses during operation or catastrophic events such as, for example, lightning. 

As a consequence, the degradation through erosion, starting with the first symptoms and up to the complete destruction of the blade material is investigated, looking to find how the composite layers are destroyed and how the resistance to abrasive wear can be improved. The final goal of this work is to identify how the blade material’s degradation occurs and evolves. Also, some constructive solutions that allow the prevention of degradations or the slowing down of their evolution are investigated.

## 2. Materials and Methods

### 2.1. Tested Material Characteristics

The samples of the material used in this work are 30 × 60 mm pieces, extracted from a hybrid composite material used for an industrial-grade wind blade. The composition of the material encompasses several layers:

- Protective layer, consisting of paint and a hard polymeric material.
- Glass fiber fabric reinforcement, consisting of two layers oriented in two directions, at 45°.
- High-density foam core
- Epoxy resin base layer

Each one of these layers has its own contribution to the composite material properties, leading to high mechanical resistance under conditions of low weight. 

**Figure 1:**
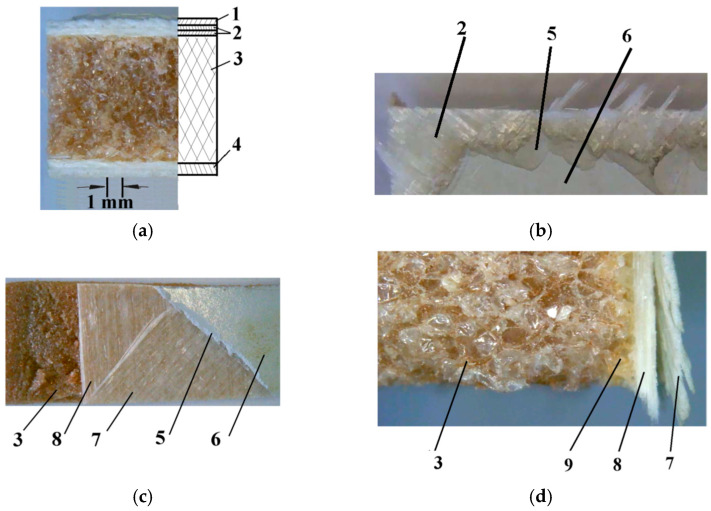
Composite structure of wind turbine blade casing: (**a**) cross section-real view/diagram; (**b**) top view; (**c**) fiberglass reinforcement detail; (**d**) core foam detail 1; outer protective layer, 2; glass fiber fabric reinforcement, 3; high-density PET core foam, 4; glass fiber fabric reinforced epoxy resin base, 5; anti-erosion protective layer, 6; paint layer, 7; top fiberglass fabric layer, 8; bottom fiberglass fabric layer, 9; adhesive layer.

In order to identify the samples’ cross-section geometric dimensions, optical measurements were performed, following methods described by Sawyer et al. [15]. For this purpose, samples were cut from the wind blade, using a disk saw, at low rotation speed in order to avoid thermal modification of the material, and an optical microscope equipped with a digital camera (Microscope Optika OPSZN-9, produced by Optika s.r.l., Ponteranica, Italy, equipped with digital camera S3CMOS, USB3.0, 1280X960, produced by Touptek Photonics, Zhejiang, China) was used for digital images of cross-section acquiring. After scale establishment (Figure 1a) the measurements were performed on the digital images, as recommended Wu et al. [16], using the Digimizer software (Digimizer 4.6.1, Copyright 2005-2016 MedCalc Software Ltd., Ostend, Belgium). Figure 1 presents the physical aspect of the samples. There are visible the composite’s layers and the way that these are overlapping. In order to fix together the component layers, an adhesive solution is used. After optical analysis and measurement of the material samples, the corresponding layers’ dimensions were found. These are presented in Table 1.

**Figure 2:**
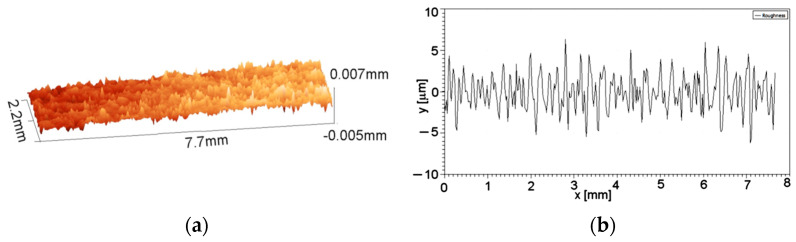
Samples’ surface characteristics: (**a**) 3D profile; (**b**) roughness graph.

Following Zbynek et al. [17], in order to establish the surface topography, some appropriate measurements must be performed. Using a 3D profiler, the samples’ surfaces were analyzed, Figure 2 and roughness parameters were measured, Table 2.

As can be seen in Table 2 and according to Hair et al. [18], the values for skewness and kurtosis show that the measured values are valid.

### 2.2. Abrasive Material Characteristics

**Figure 3:**
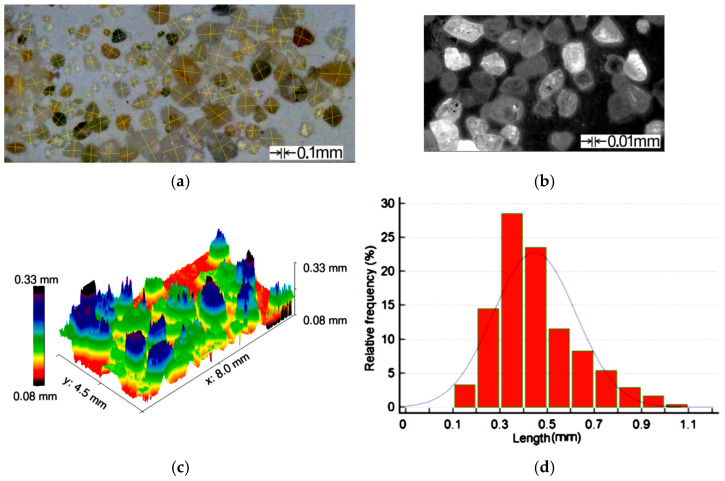
Sand particles: (**a**) dimensions; (**b**) optical analysis; (**c**) 3D profilometry; (**d**) statistical distribution of particles’ dimensions.

Following similar tests conducted by other researchers, Mishra et al. [19] used common sand as abrasive material, with the particles’ dimensions between 0.2 and 0.8 mm. The obtained results are presented in Figure 3.

Figure 3 presents the characteristics of the abrasive particles. In order to measure the abrasive particle dimensions, a method based on optical microscopy, following the Raadnui [20], was used together with the Digimizer computer software (Figure 3a). An important aspect regarding the abrasive capacity of the sand particles relies on their sharp edges. With the aim to investigate the shape of the particles, an optical 3D profiler was used (Figure 3b). The 3D reconstruction, obtained with Gwyddion v.2.47 software package, allows us to understand how the sharp edges are positioned on the particles (Figure 3c). Since during the performed measurements, several dimensions were obtained, a statistical analysis of the particles was performed, in order to check the distribution of the dimensions in the required domain. For this purpose, 500 particles were measured, and the obtained results were processed with the MedCalc statistical package (MedCalc Software Ltd., Ostend, Belgium), in order to find the particle dimensions’ distribution between minimal-maximal measured values (frequency). The results show that more than 28% of particles have dimensions between 0.3 and 0.5 mm, Figure 3d. This means that the used abrasive material complies with the initial requirements.

### 2.3. Abrasive Erosion Testing Method

The erosion test methodology differs, in the case of composite materials used for wind turbine blades preferred the erosion method with hard particles carried in the air stream, Wood et al. [21]. This method is in accordance with the ASTM G76 standard [22], assuming the entrainment of the abrasive particles in a stream of pressurized air, towards the test specimen and measuring the resulting effects by the amount of material removed or by assessing the degree of destruction of the sample surface.

**Figure 4:**
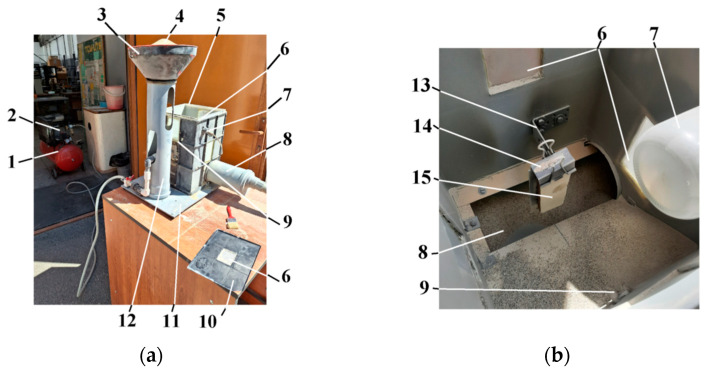
Air Erosion Test Rig; (**a**) General view; (**b**) Testing area; 1—air compressor; 2—pressure control device; 3—abrasive material tank; 4—abrasive material; 5—testing enclosure; 6—observation windows; 7—illumination system; 8—used abrasive collecting system; 9—calibrated nozzle 4.9mm; 10—enclosure cover; 11—tank support; 12—base plate; 13—sample support; 14—sample fixing clamp; 15—sample.

The testing principle Is presented In ASTM G76. Different researchers use test rigs with different structures but respect the main principles recommended by the standard. As a consequence, in order to complete the present work, a test rig was designed and built, Figure 4. The test rig is equipped with a pressurized air source, pressure control devices, and abrasive material feeding system.

In the present work, the effect of erosion was studied at a qualitative level, analyzing the structural degradation of the samples subjected to air streaming of abrasive particles. The degradation was evaluated by the measurement of the depth evolution through the material layers, according to the applied condition of the tests. 

In order to analyze the evolution of the wear degradation the test conditions were established, namely the distance between the nozzle and the sample, the angle of impact between the abrasive jet and the sample, and the exposure time to the abrasive action. 

Given that only the appearance and evolution of the degradation are analyzed, test conditions were chosen that way to lead to an accelerated erosion, starting from a few scratches on the sample surface layer and ending with the complete destruction of the samples.

In order to establish the control of the speed value, Equation (1) (Hutchins et al. [23]) was used.

V^2^ = (k · P_a_)/(d^0.57^ · ρ_a_^1.08^) (1)

where:

k = correction coefficient (7900); P_a_ = air pressure (kPa); d = abrasive particles dimensions (µm); ρ_a_ = particles density (Mg/m^3^).

Taking into account that, due to the distance between the nozzle’s exit and the sample, the particles’ speed value at the impact with the surface of the sample is lower, a correction on the results obtained from Equation (1) must be applied, according to Wensing H. [24]. Figure 4a presents the influence of the distance between the nozzle and sample on the particles’ speed value evolution from the nozzle to sample and Figure 4b presents the influence of the air stream pressure on the particles’ speed values, measured both on the sample’s surface and at exit from the nozzle. 

**Figure 5:**
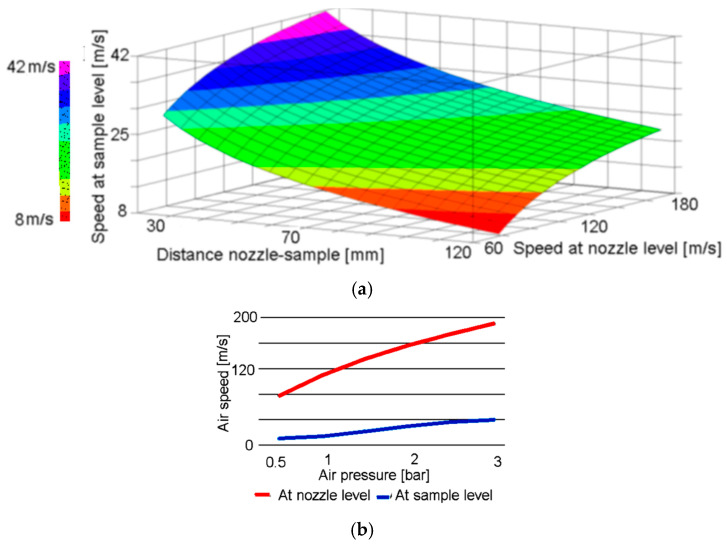
Abrasive particles’ speed evolution: (**a**) depending on distance between nozzle and sample; (**b**) depending on the air stream pressure.

According to Figure 5, the particles’ speed can be controlled by modifying the nozzle-sample distance, or by modifying the air stream pressure. In this work, the distance was kept at a constant value and the particles’ speed variation was obtained through air pressure modification.

Looking for the evaluation of degradation produced by erosion and a clear separation of the effects on each layer, the tests’ parameters must be established. Since accelerated erosion is targeted, the impact angle between the erosive agent and the sample’s surface is kept at the worst value (90°) and the nozzle-sample distance at 120 mm, following the recommendation of Satapathy et al. [25]. These conditions were met with the special holder of the test rig, which allows quick mount-unmount of the sample with the help of an elastic metallic clamp.

In order to establish the values for particles’ speed and exposure time, some preliminary tests were performed. Following the obtained results, two values were chosen for speed: 14 m/s and 30 m/s, and two values for exposure time: 90 s and 180 s Combination of these values leads to the obtaining of barely visible scars on the sample surface or to the total destruction of the tested material. Based on all the above presented, the erosion tests’ conditions were established, Table 3.

As can be observed in Table 2, there are two parameters that can be modified during the tests: the particles’ speed and the exposure time. Different combinations of the values for these parameters allowed the obtaining of several degradation degrees.

For each combination of testing parameters, five samples of chosen material were tested, and the corresponding obtained degradation depth values were averaged. The resulting value was used in further analysis. 

### 2.4. Artificial Neural Network Modeling

In order to establish which is the most important parameter leading to the degradation process, a method based on Artificial Neural Networks (ANNs) modeling is proposed. The ANNs are parallel computing systems made up of elementary units (artificial neurons), organized in complex structures and interconnected through information channels controlled by transfer functions, Gurney [26]. ANN-based modeling requires only known input-output datasets, without the need for other mathematical dependencies between the modeled data. This way offers the possibility to predict the evolution of the modeled phenomena or establish the hierarchy of the input’s importance over outputs only based on the experimental acquired datasets, Thakur et al. [27]. This behavior makes them particularly useful in modeling phenomena for which there are available only experimentally acquired datasets, whiteout the knowing of the corresponding mathematical formulas existing between the data. 

There is also a drawback in using ANN-based modeling: the obtained results are assumed to have a certain error, both in the training stage and in the validation stage, these values are limited and accepted by the user, Chen et al. [28]. These errors can be evaluated using several methods, in this work, the Pearson’s correlation coefficient R was used in order to establish the corresponding error’s value during the training stage and MAE for the validation stage, following the indication of Marin in [29]. There are several ANNs’ architectures, meaning the number of neurons and layers, but the most used for prediction and hierarchy analysis is the Feed-Forward one, as Nazare et al. recommended in [30]. In this type of network, the information travels only from the inputs to the outputs of the ANN.

The modeling procedure applied in this work requires the ANN’s structure (this one is tightly linked to the processed dataset), the network training using available data looking for a prescribed error, and the use of the model to investigate the inputs (particles’ speed and exposure time) importance over output—degradation depth. In order to investigate the possibility of improving the blade material resistance against the erosion phenomena, some tests were performed with samples having the surface protected by applying an industrial grade polymeric adhesive foil, with high resistance in abrasive wear conditions properties.

## 3. Results

### 3.1. Erosion Tests’ Results

During the tests performed, the evolution of abrasive wear degradation was monitored by degradation depth measuring. Figure 5 presents the results obtained for the lowest particle speed and testing time (particles’ speed = 14 m/s, time = 90 s).

**Figure 6:**
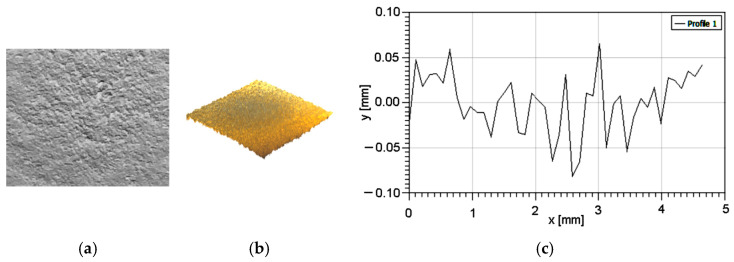
Degradation by erosion at v = 14 m/s and t = 90 s: (**a**) optical image; (**b**) 3D profilometry; (**c**) section through degraded area.

One can observe in Figure 6 that the only affected is the paint layer, showing scars with partial penetration, with a depth value of 0.085 mm. Also, are present some particle fragments with dimensions around 0.06 mm. These particles are attached to the sample surface.

**Figure 7:**
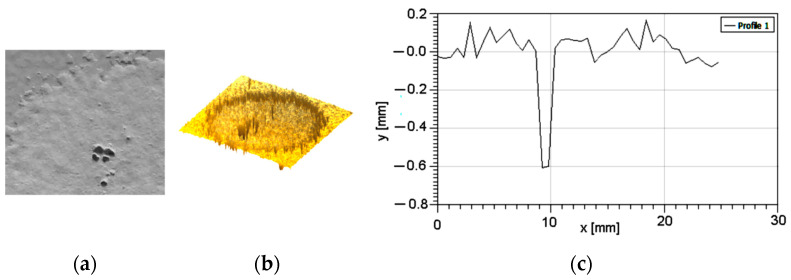
Degradation by erosion at v = 14 m/s and t = 180 s: (**a**) optical image; (**b**) 3D profilometry; (**c**) section through degraded area.

Figure 7 presents the results obtained after testing the samples at particles’ speed = 14 m/s and exposure time = 180 s. In this case, a total penetration of the paint layer can be observed, with a partial penetration into the protection layer.

**Figure 8:**
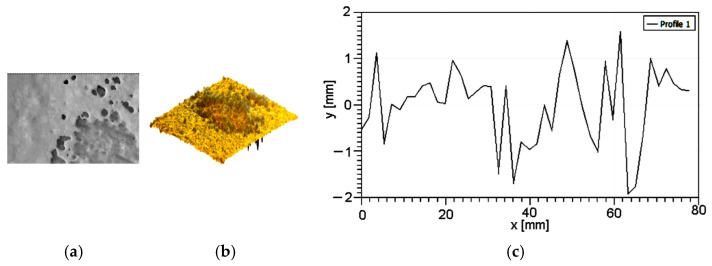
Degradation by erosion at v = 30 m/s and t = 90 s: (**a**) optical image; (**b**) 3D profilometry; (**c**) section through degraded area.

Figure 8 presents the results obtained after the testing with particles’ speed = 30 m/s and the value of exposure time = 90 s. Can be observed that the degradation is higher, the cover being totally penetrated, leaving exposed the glass fabric layers. These layers are also partially penetrated and reach the 0.5 mm depth in core foam. The total degradation depth has a value of 1.9 mm.

**Figure 9:**
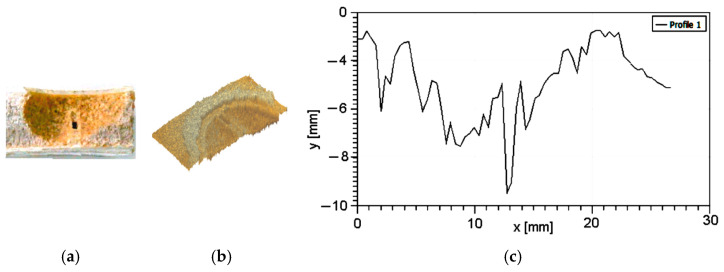
Degradation by erosion at v = 30 m/s and t = 180 sec: (**a**) optical image; (**b**) 3D profilometry; (**c**) section through the degraded area.

Figure 9 presents the results obtained for testing at particles’ speed = 30 m/s and exposure time = 180 s.

In Figure 9, it can be observed that all layers are penetrated, until the bottom one—the epoxy resin base. The degradation of the material is complete, the core foam is not only penetrated but totally removed from the sample.

After the tests were performed some observations were available, these are presented in Table 4.

**Figure 10:**
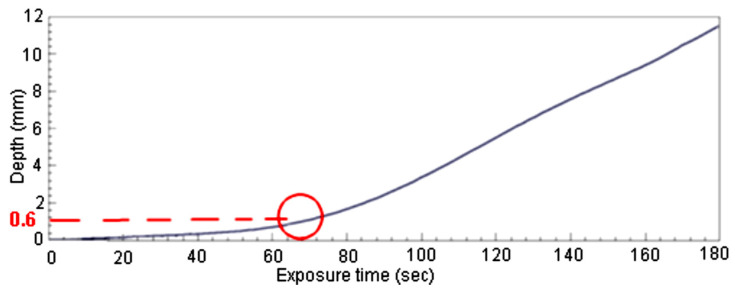
Wear degradation depth evolution after surface layer penetration, for speed = 14 m/s.

Following the tests results came out that the surface layer has the most important role in the protection of the blade against abrasive wear. Figure 10 presents the evolution of the depth during the erosion process depending on exposure time, computed from the tests’ results. After exceeding the cover layer thickness (paint layer 0.084 mm + protection layer 0.518 mm = 0.602 mm, marked with red dashed line), the degradation depth starts to increases rapidly, see the red circle. This leads to the conclusion that the only effective protection against abrasive degradation is provided by the cover layers. As a consequence, in order to obtain high erosion resistance, the surface layer of the wind blade must be carefully protected.

### 3.2. ANN Modeling Results

In order to investigate the importance of particles’ speed and exposure time on the degradation evolution, an ANN-based model was built, with input particles’ speed (Speed) exposure time (Time), and degradation depth (Depth) as output. 

Taking into account that the ANN architecture is highly dependent on the processed data, an optimization procedure is required. As a consequence, in order to establish the optimal architecture of the RNA model, based on experimental data acquired after performing the erosion tests, the genetic algorithm-based Pythia software (Pythia 1.02, Copyright Runtime Software) was used, following Haidan et al. [31], with the appropriate settings presented in Table 5.

After obtaining the ANN architecture, based on the genetic algorithm performing, the next step is to train the network, choosing the specific settings, this set of properties being the optimal ones for analyzing the abrasion degradation phenomena evolution Zucatelli et al. [32]. As training error and validation error, R > 0.999 and MAE < 0.8 values were chosen, Chai et al. [33]. These values are presented in Table 6. As training data were used the experimentally acquired values for input-output categories, some examples being presented in Table 7. 

For ANN modeling and performing, the Neural Power software (Neural Power, Professional version 1.64, Copyright 1997-2002, CPC-X software) was used, Marin et al. [34] was used, since this one allows both training and validation of the ANN, with the user’s prescribed errors. 

**Figure 11:**
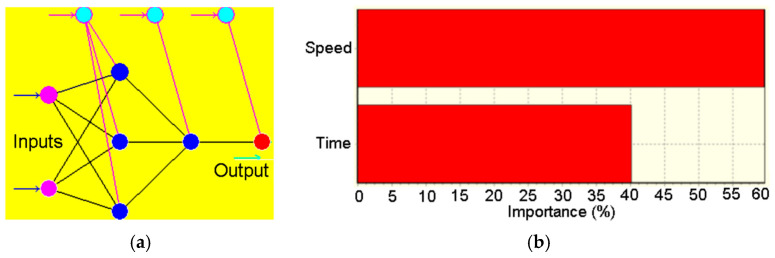
ANN model: (**a**) architecture; (**b**) inputs importance over output. Color code: Magenta—input nodes; Dark Blue—hidden nodes; Red—output node; Light Blue—bias nodes.

Figure 11a presents the obtained ANN architecture and Figure 11b presents the importance hierarchy obtained after model running.

As can be observed in Figure 11b, the most important parameter influencing the degradation depth is the particles’ speed, with 20% more than the exposure time. 

**Figure 12:**
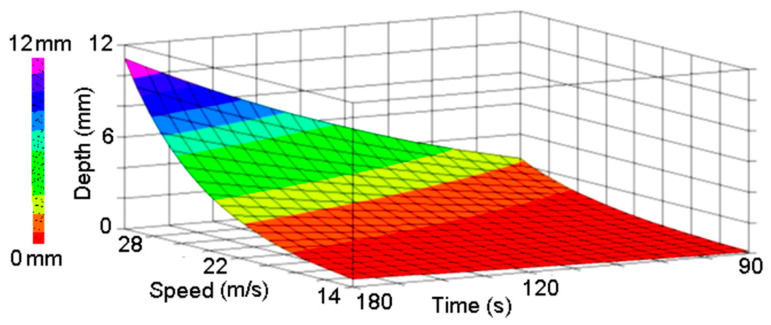
Wear degradation evolution during erosion tests.

Based on the obtained results after the tests’ performance, the available data allowed us to obtain a regression equation (Equation (2)) and a corresponding evolution graph for degradation depth under the influence of particles’ speed and exposure time, Figure 12.

Y = a∙(b^Speed^)∙(c^Time^) (2)

where:

a = 4.1683 × 10^−7^; b = 1.2073; c = 2.2037.

### 3.3. Protection Foil Tests’ Results

**Figure 13:**
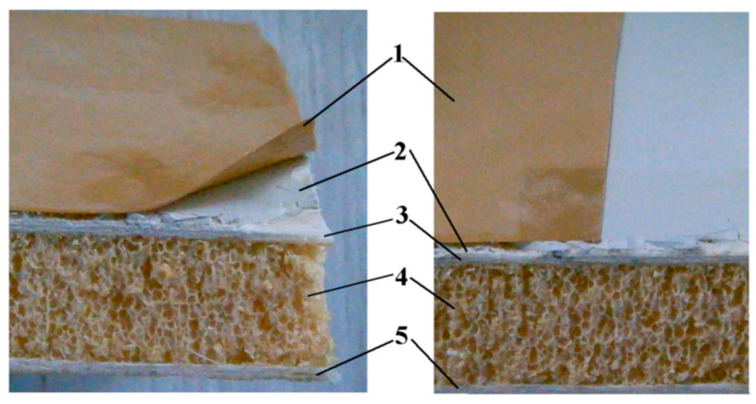
Protected sample 1; foil, 2; outer layer, 3; glass fiber layer, 4; PET foam, 5; epoxy base.

In order to improve the wind blade material protection, a foil with anti-erosion properties was applied to the outer surface, Figure 13.

**Figure 14:**
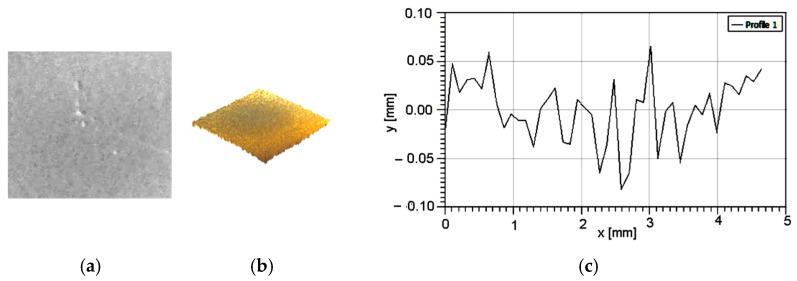
Degradation by erosion at v = 30 m/s and t = 90 s: (**a**) optical image; (**b**) 3D profilometry; (**c**) section through degraded area. Surface protected with foil.

To assess the effectiveness of the method, samples with applied foil were tested. Since at the particles’ speed of 14 m/s and for an exposure time of 180 s no changes were observed on the surface protected with the foil, the tests were carried out only at the particles’ speed of 30 m/s. Figure 14 presents the degradation obtained for the testing with an exposure time value of 90 s. The depth has a value of 0.01 mm; meanwhile, during the tests in the same conditions but without foil, the depth value was 2.1 mm.

**Figure 15:**
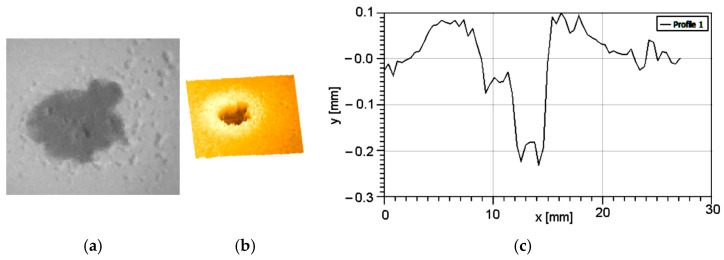
Degradation by erosion at v = 30 m/s and t = 180 s-surface protected with foil: (**a**) optical image; (**b**) 3D profilometry; (**c**) section through degraded area. Surface protected with foil.

In Figure 15 are presented the results obtained after the testing with 180 s exposure time. The degradation depth value was 0.816 mm (0.25 mm penetration into the core foam) meanwhile without protection the degradation depth value was 10 mm.

The comparison between the depth measurement results obtained after testing with and without protective foil allowed the computing of the protection method efficiency, according to Equation (3).

Efficiency = ((Depth without foil − Depth with foil) × 100)/Depth without foil
(3)

Table 8 presents the numeric computed values of degradation depths and of the efficiency, in the case that the protective foil is applied.

Looking into Table 8, it is obvious that the protective method, based on the use of an erosion-resistant foil applied on the blade surface, leads to an improvement of the material resistance to the action of aggressive factors, like abrasive particles, with 95.68%, comparing with the unprotected material. Another advantage offered by the use of protective foil is the possibility of the early establishing of the appearance of the initial degradation, due to the color difference between the foil and composite material’ surface. This establishment can be obtained by optical inspection of the wind blade.

## 4. Discussion

The aim of this work was to investigate the initiation and evolution of degradation that occurs when the blade of a wind turbine’s propeller is working in aggressive environments, where abrasive particles are present. Due to the high rotational speed of the propeller and wind speed, the impact with the particles leads to degradation of the blade material, Hasager et al. [35]. Taking into account that the wind blades are made of multi-layered composite materials, the erosion process effects are scars, more or less deep into the composite layers, as other research has shown, Patnaik et al. [36], Srivastava et al. [37].

In order to accomplish the analysis proposed, a test rig was designed, based on general methodologies used for composite materials testing, Carlsson et al. [38] and complying with the requirements of the ASTM G76 standard. The tested samples were extracted from an industrial-grade wind turbine propeller blade, made of a composite polymeric material. The initiation and evolution of erosive wear was established by measurements of the degradation depth into the material layers. 

Following the results obtained after tests performed, was found that the erosive agent, such as sand, existing in the operating environment of the wind turbine propellers stands as the main aggressive factor, leading to wind blade degradation. Since the materials used are layered composites, this degradation affects first time the surface layers, penetrating into the deeper ones, this observation is in good concordance with others’ scholarly findings, Aird et al. [39]. 

In order to analyze the influence of erosive process parameters (the particles’ speed and exposure time) an ANN-based model was built since this method proved to be appropriate for this type of investigation, Suresh et al. [40], Carta et al. [41]. For the generation of the ANN structure a genetic algorithm was used, ensuring the optimal concordance with the experimental acquired datasets. Performing an analysis on the influence of the particles’ speed and the exposure time on the evolution of the degradation process, was found that the most influencing parameter is the particles’ speed, exceeding with almost 20% the exposure time’s influence.

In the case of the analyzed composite material, was found that the most effective protection against the environmental erosive action is the superficial layer, as other researchers also mentioned by Keegan et al. [42]. After the penetration of this one, the degradation depth increases rapidly, leading to the total destruction of the material.

Since the wind blade’s surface degradation leads to a decrease in its performance, as Wang et al. [43] shown, in order to improve the wind blade material’s resistance to abrasive processes was proposed a method based on the applying, to the surface of the material, of protection consisting into an erosion resistant foil. The samples extracted from protected material were tested in the most aggressive conditions. Was found that the method leads to an increased resistance to the erosive processes with 95.68% compared with the unprotected surface.

## 5. Conclusions

Comparing with the previous research, performed by several scholars (Thomas et al. in [6], Dathu et al. in [13], etc.), targeting mainly the design of new composite materials recipes for wind blades, with the aim to improve erosion resistance, in the presented work a real industrial blade composite material was tested. This material is made of several layers, each one adding specific properties to the whole. In order to investigate how the material’s failure occurs in case of erosion wear, the tests were performed in the most aggressive conditions, using sand abrasive particles. The structure of the material was previously investigated, in order to identify the layers and their composition. Was found that there are three layers: a cover, made of a rigid polymer, a second one, made of two glass fibers fabric, oriented at 45°, a thick polymeric foam layer, and a base layer, made of epoxy resin. These layers are glued together, resulting in the hybrid composite material of the wind blade. 

Some researchers studied the wear resistance only of the cover layer (Zhang et al. in [7], Mishnaevsky et al. in [9], Slot et al. in [10], etc.). The results obtained in the presented work are consistent with their findings, showing that the most efficient protection against erosive wear is the cover layer. Testing the erosion resistance with air-born sand particles, at high-speed values, reaching 30 m/s (108 km/h) and exposure times reaching 180 s, allowed us to identify the damage evolution, from the very little scars, with a depth value of 0.085 mm, until the total penetration. In addition to this, was investigated how the inner layers of the material react after the cover layer penetration occurs. Was found that the inner layers are far less resistant to erosion. After the cover layer destruction, at a depth value of 0.6 mm, the degradation speed increases rapidly, further exposure to the abrasive agent leading to the total failure of the material, the erosion generated hole reaching the bottom epoxy resin layer, at 11 mm from the upper cover layer.

In order to identify which factor- speed or exposure time, is more important, during the erosive process, an artificial neural network-based model was built. Was found that the most influencing factor is the particle speed, with about 20% more compared with the exposure time. These findings are in good concordance with the results obtained by other researchers (Suresh et al. in [40]).

Following the obtained results, a protection method for abrasive wear improvement was proposed, consisting of a polymeric foil applied on the cover layer. Performing several erosive tests on the protected samples, results showed an increase in the protection efficiency by more than 95%, compared with the unprotected samples.

Overall, the presented work presents the novelty of testing real industrial-grade wind blade material against abrasive erosion, clarifying how the degradation evolves, and which is the most important factor—abrasive particle speed. Also, a protection method was proposed and validated.

## Figures and Tables

**Table 1:** Tested samples’ layers dimensions.

Paint (mm)	Protection (mm)	Glass Fibers (mm)	Glass Fiber Diameter (mm)	Foam (mm)	Base Epoxy (mm)
0.084	0.518	upper layer = 0.318 lower layer = 0.389	0.025	10	2

**Table 2:** Samples’ surface roughness values (µm).

Profile Length	Amplitude Ra	Roughness Average Rq	Skewness Rsk	Kurtosis Rku
7720	1.8	2.2	0.018	2.94

**Table 3:** Abrasive erosion testing conditions.

Particle Speed (m/s)	Sample-Nozzle Distance (mm)	Impact Angle (°)	Testing Time (s)
14; 30	120	90	90; 180

**Table 4:** Abrasive erosion testing observations.

Particle Speed (m/s)	Testing Time (s)	Degradation Depth (mm)	Observations
14	90	0.085	paint partial penetration
	180	0.6	protection layer partial penetration
30	90	1.9	foam penetration 0.5 mm
	180	11	foam penetration 11 mm

**Table 5:** Genetic algorithm settings.

Criteria	Value	Generation Specifications	Generation
Root mean square deviation Maximum squared deviation Maximum number of neurons	0.001 0.1 100	Individuals number Generation number Mutation rate Crossover rate Individuals selected	50 1000 0.04 0.02 10

**Table 6:** ANN training settings.

Network Type	Transfer Function	Training Algorithm	Training Rate	Momentum	Training Error Limit	Validation Error Limit
Feed-forward	Tanh(x)	90	0.15	0.8	R > 0.999	MAE < 0.8

**Table 7:** ANN training data examples.

Input 1 Speed (m/s)	Input 2 Time (s)	Output Depth (mm)
14	90	0.09
….	….	….
14	180	0.6
….	….	….
30	90	1.9
….	….	….
30	180	11

**Table 8:** Depth and efficiency values.

Time (s) Depth (mm)	90	180
Without foil	2.1	10
With foil	0.01	0.816
Difference	2.09	9.184
Efficiency (%)	99.52	91.84
Average efficiency (%)	95.68	

## Data Availability

The raw data supporting the conclusions of this article will be made available by the authors upon request.
